# Engaging Mentor Mothers in Rapid Return of Viral Load Results to Pregnant and Postpartum Women Living with HIV: An Implementation Pilot Study

**DOI:** 10.1007/s10461-025-04987-2

**Published:** 2025-12-23

**Authors:** Pamela M. Murnane, Sharon Ouma, Raphael Onyango, Nita Mukand, Isabelle Thapar, Francesca Odhiambo, Jane Kabami, Elizabeth A. Bukusi, Craig R. Cohen, James Ayieko

**Affiliations:** 1 Department of Epidemiology & Biostatistics, University of California San Francisco, San Francisco, USA; 2 Institute for Global Health Sciences, University of California San Francisco, 550 16th Street, San Francisco, CA 94158 USA; 3 Center for Microbiology Research, Kenya Medical Research Institute, Nairobi, Kenya; 4 Infectious Diseases Research Collaboration, Kampala, Uganda; 5 Department of Obstetrics, Gynecology and Reproductive Sciences, University of California San Francisco, San Francisco, USA

**Keywords:** HIV, Pregnancy, Mentor mothers, Viral load monitoring, Adherence, Women

## Abstract

In Kenya, the primary driver of perinatal HIV transmission is disengagement from care. Virologic monitoring can reinforce adherence and enable timely intervention, yet results are often delayed. Among pregnant (≥ 27 weeks) and postpartum (≤ 6 months) women in HIV care in Kisumu Kenya, we conducted a pilot study in which Mentor Mothers (peer support) returned viral load results (GeneXpert) after a clinic visit between June-December 2023. We assessed feasibility, acceptability, and factors associated with time from sample collection to results to participants via generalized estimating equations with log-transformed time as the outcome and report geometric mean ratios (GMR). We assessed the potential impact on viremia after 6 months using two control groups (one retrospective, one prospectively enrolled). Among 545 participants (273 pilot,123 prospective and 149 retrospective controls), the median age overall was 30 years (interquartile range [IQR] 26–35) and 40% were pregnant. In the pilot, Mentor Mothers returned 100% of 422 viral load results of which 90% were suppressed. The median time from sample collection to result return was 27.0 h (IQR 23.3–54.6). We observed a trend towards slower times among women with prior viremia (adjusted-GMR 1.22, 95%CI: 0.98–1.52). The odds of subsequent viremia did not differ between pilot and control groups (adjusted-OR 1.30, 95%CI: 0.71–2.38). Mentor Mothers reported this approach was a good use of their time. In conclusion, Mentor Mothers rapidly delivered viral load results though we did not observe an impact on viremia. This approach could complement other strategies to optimize person-centered care for pregnant and postpartum women.

*Trial registry* NCT05845619.

**Supplementary Information:**

The online version contains supplementary material available at 10.1007/s10461-025-04987-2.

## Introduction

With near universal access to antiretroviral treatment (ART) across sub-Saharan Africa, perinatal HIV transmission has significantly declined in recent decades. However, few countries have been able to reach elimination targets [1, 2]. In Kenya the primary driver of ongoing perinatal HIV transmission is disengagement from care and treatment [1]. Pregnancy and the postpartum period bring new responsibilities and changing routines that may present challenges to treatment engagement. On the other hand, given the risk of perinatal transmission, women are often highly motivated to protect their infant [3], yet may lack the resources or knowledge necessary to maintain virologic suppression and care engagement [4, 5]. Virologic monitoring and counseling empowers women with knowledge of their suppression status and enables intervention for those with viremia [6]. 

Kenyan guidelines for virologic monitoring for pregnant and breastfeeding women living with HIV include viral load assessment at the first antenatal care visit if already on treatment or three months after antiviral initiation, followed by 6-monthly monitoring until the end of breastfeeding [7]. Blood samples are typically sent to centralized national laboratories for testing and due to limited resources, results are returned to health facilities within weeks, or sometimes months [8]. In the event of an unsuppressed viral load result, patients are promptly contacted and provided enhanced adherence counselling, while suppressed results are routinely not returned to patients until the following clinic visit. These long wait times, coupled with infrequent monitoring, represent missed counseling opportunities to intervene early in the presence of viremia and to reinforce good adherence for those who are suppressed.

Point-of-care viral load monitoring is a promising approach to address long wait times [9], yet data on implementation strategies are limited. Current point-of-care technologies do not require specialized laboratory staff for sample processing and costs are comparable, if not lower, than sending samples to centralized laboratories [10, 11]. However, sample processing still takes well over an hour and requires space and ongoing system maintenance [9]. It is unlikely such systems will be placed at every small or remote health facility in resource limited settings. Even in facilities co-located with a point-of-care system, the time between sample collection and result return to the provider may be too long to encourage patients to wait, particularly in busy, crowded clinics. Thus, result-tailored counselling during the clinic visit may not be feasible with current technology, and provision of results will still likely require post-visit contact in most routine settings.

The Kenya Mentor Mother Program trains women living who have recently received prevention of perinatal HIV transmission services to provide peer support and adherence counselling to other pregnant and breastfeeding women living with HIV [12]. Given their established role as the primary health facility support for this population, we engaged Mentor Mothers to ensure rapid turnaround time of viral load results run on the GeneXpert platform in a pilot study in Kisumu Kenya. We hypothesized this approach would be feasible, acceptable, and could potentially reduce the risk of subsequent viremia for pregnant and postpartum women.

## Methods

### Study Setting and Sample

Participants were recruited at four Ministry of Health facilities in Kisumu County, which is one of the highest HIV burdened areas of Kenya with 21% HIV prevalence among women aged 15–49 years in 2018 [13]. Eligibility included: either ≥ 27 weeks or ≤ 6 months postpartum, living with HIV, and had initiated antiretroviral treatment. We focused on late pregnancy and the early postpartum period because of the high-risk of HIV care disengagement during this time [14]. This study includes three groups: (1) controls retrospectively sampled from records who met eligibility criteria between August and October 2022; (2) controls prospectively enrolled during May 2023; and (3) pilot study participants who received the intervention between June and December 2023.

### Intervention Description

Mentor Mothers, employed by the Kenyan Ministry of Health, include women who are living with HIV who have recently received prevention of perinatal HIV transmission services, have disclosed their HIV status to at least one household member, and have at least primary level education to ensure literacy. Training for the role includes all aspects of prevention of perinatal transmission, antenatal care, and counselling techniques to address psychosocial challenges [12]. The pilot intervention was implemented over a 6-month period and included three components: (1) rapid (as soon as possible) return of all viral load results via trained Mentor Mothers; (2) viral load monitoring at baseline and after 3 months for those with sufficient follow-up time during the six-month pilot period; and (3) enhanced counselling regarding low-level viremia (detectable up to 1000 copies/mL). We provided Mentor Mothers a laminated color copy of a graphical counseling aid depicting the reduction of HIV in blood when antiretrovirals are present,, which was developed and used successfully in prior work in Uganda [15]. 

### Study Procedures

Recruitment, enrollment, and the baseline study visit were identical for prospectively enrolled controls and pilot participants. Study staff visited each facility at least one day per week to recruit participants, opting for the day with the highest patient flow. They worked closely with Mentor Mothers to recruit all eligible participants attending clinic on recruitment days. In a private space at the clinic, study staff administered written informed consent, enrolled women, and administered questionnaires and collected a blood sample for viral load testing. The demographic and clinical questionnaire is included in the Supplementary Materials. We also administered the Perceived Stress Scale [16], the Edinburgh Postpartum Depression Scale [17], and the Household Food Insecurity Access Scale [18]. On rare occasions we enrolled participants at home if time did not permit completion of the interviews at the clinic. Home visits were facilitated by Mentor Mothers who first contacted potential participants to assess their interest in the study and willingness to have a home visit. Our staff also ensured confidentiality by not disclosing the purpose of their visit to others in the community or home, and arriving by means of local transit. All data was captured directly into REDCap [19, 20]. 

One facility had an existing Cepheid GeneXpert platform and served as the testing lab for this study. Blood samples were brought to the lab on the day of collection for testing via the Xpert HIV-1 Viral Load assay (lower limit of quantification: 40 copies/mL) [21]. Samples brought late in the day were centrifuged and the plasma was frozen for testing on the next work day. Viral load results for prospective controls were returned to patient files in accordance with standard of care. During the pilot study period, results were promptly returned to Mentor Mothers via phone, and Mentor Mothers, in turn, promptly phoned study participants with results. We used a paper tracking form to document the time from sample collection until the result was delivered to the participant, noting the time for each step in the process. We tracked the time of sample collection, sample delivery to the lab, completion of GeneXpert processing, return of results to study team from the lab, and the time the study team contact the Mentor Mother. The Mentor Mothers documented the time they reached the participant on a separate form where they also documented the number of attempts made to reach each participant, the method of contact, and any referrals made for psychosocial, nutrition, or other services. Mentor Mothers received a reimbursement of 300 Kenyan Shillings (~ USD $2.30) for each participant reached to cover effort and phone airtime. Our Study Coordinator met with Mentor Mothers on a weekly basis throughout the pilot study period to collect their documentation, discuss any challenges, and encourage open communication. We did not systematically validate that participants were reached at the times indicated by the Mentor Mothers.

Retrospective controls were identified through paper-based registers (antenatal care and HIV-exposed infant registers) at each of the four facilities and were not contacted for data collection; only de-identified data were collected. Among those eligible during a hypothetical enrollment period of August through October 2022 (*N* = 314) we randomly sampled 150 to serve as controls and linked clinic IDs to electronic records. Their assigned “enrollment” date was their first clinic visit recorded in the electronic medical record system between August and October, or September 15th if no visit was recorded.

We merged study data with de-identified electronic medical records for clinic visit history and most recent ART regimen and with the National AIDS & STI Control Program (NASCOP) viral load records one year prior to enrollment and up to ten months after enrollment.

### Study Outcomes

Our primary outcome was the time required for viral load processing and result management, from viral load sample collection to return of results to the participant, as documented by the study team and Mentor Mothers. Additionally, we report the proportion of women with viremia who received referrals for enhanced support services. We also administered surveys to eight Mentor Mothers six month after the pilot with four questions adapted from the Clinical Information Systems Success Model [22] to assess feasibility, eight questions adapted from the Evidence-Based Practice Attitude Scale [23] to assess acceptability, and one open-ended question about overall impressions of the intervention. Because we only asked one open-ended question, we did not employ formal qualitative analysis methods; rather, we summarized common answers.

To assess the potential impact of the intervention on subsequent viremia, we first subset all NASCOP viral load results to those collected between 2 and 10 months after study enrollment, then, if more than one viral load was collected during that period, we selected the measure closest to 6 months (180 days) following enrollment. Viremia was defined as > 50 copies/mL as the assays in routine care varied and this was the lower limit of detection for many of the tests.

### Analysis of Time Spent

We estimated the time spent overall and on each step from viral load sample collection to return of results to the participant. We then assessed potential predictors of the overall time, focusing on factors that could be associated with a participant’s ability or willingness to be reached promptly. These included *maternal characteristics*: age, currently pregnant or postpartum, education, depression (via the Edinburgh Postpartum Depression Scale [17]) and stress (via the Perceived Stress Scale [16]); *household characteristics*: disclosure to partner, living with partner, change of residence in the past two years, household size, and food insecurity (Household Food Insecurity Access Scale [18]); *clinical characteristics*: number of clinic visits attended in the last 12 months, history of viremia in the prior year, and the study GeneXpert viral load result; and *structural characteristics*: travel time from home to clinic, day of the week of sample collection, and whether the enrollment clinic was co-located with the GeneXpert System or not. To assess associations between these factors and time from sample collection to result return to participants, we used linear regression with log-transformed time as the outcome and accounted for clinic-level clustering with robust variance estimation [24]. We exponentiated coefficients to report geometric mean ratios (GMR). All variables with *p* < 0.20 in bivariate analyses were included in a multivariable model.

### Analysis of Subsequent Viremia

Because pilot and control groups were not randomly assigned, we considered all the potential predictors of time discussed above as potential confounders with the exception of the weekday of sample collection. Additionally, rather than a binary variable for clinic co-location with GeneXpert we included dummy variables for each clinic. We also considered whether the participant was on a dolutegravir-based regimen and years since HIV diagnosis and ART initiation. We included dolutegravir because it is associated with viral suppression and HIV clinics have been switching people to this preferred regimen over time. Because available covariates in the retrospective control group were limited (age, pregnancy status, number of visits attended in the past year, past year viremia, regimen, and clinic), we compared pilot participants to each control group separately. For unadjusted analyses, we used logistic regression. For adjusted analyses, given the large number of potential confounders, we used STATA’s double-selection least absolute shrinkage and selection operator (LASSO) procedure for a parsimonious adjusted model [25, 26]. The double-selection methods runs LASSO regression on both the primary exposure (pilot vs. control) and outcome (subsequent viremia) separately for covariate selection, then estimates coefficients and standard errors for the primary exposure adjusted for the selected covariates. Both unadjusted and adjusted models accounted for clinic-level clustering with robust variance estimation [24]. 

### Ethics

The study protocol was reviewed and approved by the Ethics Committee at the Kenya Medical Research Institute and the Institutional Review Board at the University of California San Francisco. All prospectively enrolled participants signed written informed consent prior to participation. Consent was waived for retrospective controls as only de-identified data was used. We included this retrospective sample as a comparison group fully untouched by research, and to bolster the number of controls. Although the prospective control group had minimal contact with our study, these small interactions may have influenced care engagement.

## Results

Among 550 women enrolled, two were inadvertently enrolled as both a prospective control and a pilot participant and were excluded; one was enrolled in the pilot study and as a retrospective control, therefore we only dropped their control data as that did not include participant interaction. Thus, among 273 pilot participants, 123 prospective controls, and 149 retrospective controls (Table 1), the median age was 30 (interquartile range [IQR] 26–35) which was comparable across groups. A greater proportion of women were enrolled in pregnancy during the pilot study (47%) compared to controls (33% retrospective, 34% prospective). Past year viral suppression was comparable across groups (64%), though missingness was significant (20% overall), with the largest proportion missing among retrospective controls (25%). Psychosocial circumstances were worse among prospective controls compared to pilot study participants, with median scores of 5 for depression (IQR 3–7), 16 for stress (IQR 13–19), and 19 food insecurity (IQR 15–21) among controls, compared to 3, 6, and 14, respectively for pilot participants (IQRs: 1–5 depression; 5–10 stress; 11–19 food insecurity).

**Table 1:** Maternal characteristics at baseline by study group

	Controls			
	From records	Prospectively enrolled	Pilot participants	Total
Maternal characteristics	*N* = 149	*N* = 123	*N* = 273	*N* = 545
Maternal age	31 (27–36)	30 (27–34)	30 (25–34)	30 (26–35)
Currently pregnant	49 (33%)	42 (34%)	128 (47%)	219 (40%)
Education				
None - Primary		72 (59%)	121 (44%)	193 (49%)
Secondary/vocational - some		27 (22%)	49 (18%)	76 (19%)
Secondary/vocational - complete		15 (12%)	55 (20%)	70 (18%)
Post secondary or more		9 (7%)	48 (18%)	57 (14%)
Marital status				
Single		17 (14%)	32 (12%)	49 (12%)
Married or cohabiting		89 (72%)	209 (77%)	298 (75%)
Divorced or separated		9 (7%)	18 (7%)	27 (7%)
Widowed		8 (7%)	14 (5%)	22 (6%)
Edinburgh Depression Scale		5 (3–7)	3 (1–5)	3 (2–6)
Perceived Stress Scale		16 (13–19)	6 (5–10)	8 (5–16)
Disclosed HIV status to partner		88 (72%)	185 (68%)	273 (69%)
Live with husband/partner		74 (60%)	157 (58%)	231 (58%)
Household size		4 (3–5)	4 (2–5)	4 (3–5)
Changed residence < 2 years ago		19 (15%)	20 (7%)	39 (10%)
Food insecurity (HFIAS)		19 (15–21)	14 (11–19)	15 (12–21)
Travel time from home-clinic, minutes		40 (30–60)	45 (30–60)	45 (30–60)
On DTG-containing regimen	126 (88%)	112 (92%)	249 (92%)	487 (91%)
Number of HIV clinic visits in past year	5 (3–6)	6 (5–8)	6 (4–8)	6 (4–7)
Past year viral load				
Suppressed	94 (63%)	82 (67%)	175 (64%)	351 (64%)
Viremia (≥ 50)	18 (12%)	21 (17%)	47 (17%)	86 (16%)
No record	37 (25%)	20 (16%)	51 (19%)	108 (20%)
Study baseline viral load results (GeneXpert)				
Undetectable		112 (92%)	241 (88%)	353 (89%)
41–199		7 (6%)	14 (5%)	21 (5%)
200–999		1 (1%)	4 (1%)	5 (1%)
1000+		2 (2%)	14 (5%)	16 (4%)
Years since HIV diagnosis		6 (1–9)	6 (2–10)	6 (2–10)
Years since ART start		6 (1–9)	6 (2–10)	6 (2–9)

*ART* antiretroviral treatment, *DTG* dolutegravir, *HFIAS* Household food insecurity access scale

Among 273 pilot participants we assessed 422 viral loads and 100% of results were delivered to participants by Mentor Mothers. Among them, 380 (90.0%) samples were ≤ 40 copies/mL, and 4.5%, 1.4%, and 4.0% were 41–199, 200–999, and ≥ 1000 copies/mL, respectively. Mentor Mothers referred 21 participants to enhanced monitoring or psychosocial services, including 1 (0.3%), 1 (5%), 5 (100%), and 15 (88%) among those with viral loads ≤ 40, 41–199, 200–999, and ≥ 1000 copies/mL, respectively. The median time from sample collection to result return to the participant was 27.0 h (IQR 23.3–54.6; Table 2). The slowest step in the process was time from completion of viral load analysis in the lab to delivery of results to the study team (median 15.1 h, IQR 12.6–16.7), as processing was often completed after work hours and returned to the study team the next day. The step with the greatest variability was from the time the Mentor Mother received the results to the time she reached the participant (median 5.0 h, IQR 1.3–24.9).

**Table 2:** Time in hours from blood sample collection to participant receiving viral load results: overall, by health system-level factors, and by processing step

	Median Time	Interquartile range	Range	Geometric mean (95% CI)
Overall timefrom collection to participant, full sample:	27.0	(23.3–54.6)	[3.0–434.2]	36.9 (34.5–39.5)
Overall time from collection to participant, within subgroups defined by health system-level factors				
Day of sample collection				
Monday - Thursday	26.0	(23.1–49.4)	[3.0–434.2]	34.8 (32.5–37.3)
Friday	74.7	(72.9–97.3)	[67.2–117.4]	81.6 (76.7–86.8)
Location of sample collection in relation to GeneXpert location				
Co - located at clinic	23.3	(22.2–25.7)	[3.0–434.2]	25.2 (23.1–27.5)
Not co - located	48.2	(26.3–75.4)	[18.4–191.6]	49.1 (45.3–53.2)
Time spent within each sample processing step:				
From Sample collection to lab	1.3	(0.5–2.4)	[0.0–51.6]	1.1 (1.0–1.2)
From lab receipt to viral load analysis complete	2.9	(2.2–4.0)	[0.7–44.3]	3.3 (3.1–3.5)
From viral load analysis complete to results to study team	15.1	(12.6–16.7)	[0.0–88.0]	9.0 (7.8–10.3)
From study team to mentor mother	0.9	(0.4–2.7)	[0.0–123.6]	1.4 (1.2–1.6)
From mentor mother to participant	5.0	(1.3–24.9)	[0.0–413.6]	5.7 (4.9–6.8)

In unadjusted analyses, higher maternal stress, larger household size, and greater food insecurity were modestly associated with slower times from sample collection to maternal receipt of results, but not in multivariable analysis (Table 3). In the multivariable model, the primary factors associated with slower times were structural. Samples collected on Fridays, which were typically not returned to Mentor Mothers until Mondays, took 2.02 times as long on average than samples collected on other weekdays (95% CI for geometric mean ratio [GMR]: 1.57–2.59). Results for participants attending clinics that were not co-located with the lab took 1.81 times longer than those co-located (95% CI 1.66–1.98). In terms of maternal clinical characteristics, there was a trend towards slower times among women with a history of viremia (GMR 1.22, 95%CI 0.98–1.52), but among those with a GeneXpert study viral load ≥ 1000 copies/mL, the trend was towards faster result return but with wide variability (GMR 0.80, 95% CI 0.59, 1.08).

**Table 3:** Geometric mean ratios (GMR) to quantify differences by selected factors in average times from sample collection to participant receiving viral load results

	Unadjusted		Adjusted*	
	GMR (95% CI)	*p*-value	GMR (95% CI)	*p*-value
*Maternal characteristics*				
Maternal age, per year	0.99 (0.97, 1.02)	0.34		
Currently pregnant (vs. postpartum)	1.06 (0.77, 1.46)	0.60		
Education				
None - primary	(reference)	0.12	(reference)	0.09
Secondary/vocational - some	0.89 (0.53, 1.50)		0.87 (0.53, 1.42)	
Secondary/vocational - complete	0.89 (0.74, 1.06)		0.90 (0.77, 1.06)	
Post secondary or more	0.79 (0.64, 0.97)		0.93 (0.73, 1.19)	
Edinburgh depression scale	1.01 (0.99, 1.04)	0.16	1.00 (0.95, 1.05)	0.92
Perceived Stress Scale	1.01 (1.00, 1.02)	0.01	1.01 (0.99, 1.03)	0.25
*Household characteristics*				
Disclosed to partner	0.97 (0.72, 1.32)	0.79		
Live with husband/partner	0.93 (0.75, 1.15)	0.34		
Changed residence < 2 years ago	1.08 (0.73, 1.60)	0.57		
Household size, per person	1.06 (1.00, 1.11)	0.04	1.01 (0.96, 1.05)	0.73
Food insecurity (HFIAS)	1.01 (1.01, 1.02)	0.01	0.99 (0.98, 1.01)	0.14
*Clinical characteristics*				
Number of HIV clinic visits, past year	1.01 (0.98, 1.03)	0.42		
Past year viral load				
Suppressed	(reference)	0.14	(reference)	0.05
Viremia (> = 50)	1.34 (0.97, 1.87)		1.22 (0.98, 1.52)	
No record	0.97 (0.72, 1.32)		0.91 (0.60, 1.38)	
Study baseline viral load results (GeneXpert)				
Undetectable	(reference)	0.09	(reference)	0.001
41–999	1.12 (0.63, 1.98)		1.07 (0.67, 1.71)	
1000+	0.73 (0.47, 1.15)		0.80 (0.59, 1.08)	
*Structural factors*				
Travel time home-clinic, per 30 min	0.91 (0.83, 1.00)	0.05	0.89 (0.80, 1.00)	0.05
Sample collected Friday (vs. Mon-Thu)	2.34 (1.30, 4.23)	0.02	2.02 (1.57, 2.59)	0.003
Clinic not co-located with GeneXpert	1.95 (1.52, 2.50)	0.003	1.81 (1.66, 1.98)	0.0002

*Adjusted model includes all covariates in the adjusted column

The eight Mentor Mothers interviewed after the end of the pilot study were unanimously positive in Likert scales about the feasibility and acceptability of delivering viral load results, including that it was a good use of their time, facilities were supportive of the new responsibility, and that the process helped them support women’s adherence (Supplemental Table 1). When asked, “*Can you tell me about your experience in the pilot study and how you felt about your role delivering the viral load results?”*, all noted appreciation of the fast turnaround time of results, for example “*And the result*,* the time for viral load was to come back within one day. It was just good for us to at least know who and who needs counseling most. Yes.*” *[P009]* Additionally, some noted how the timeliness of the results facilitated early intervention, for example, *“There was one with 400 and something and we started our intervention the first thing. And then we called the mother and tell her about her results and how you feel about the results.” [P010]* This Mentor Mother went on to describe how she was able to probe the client to open up about her adherence to get to the bottom of her challenges.

Six months after enrollment, 34 (14%) of pilot study participants, 15 (11%) of retrospective controls, and 13 (12%) of prospective controls had a detectable viral load (> 50 copies/mL) in routine care (Table 4). Study group and subsequent viremia were not associated in unadjusted analyses. After adjusting for clinical variables available in both control groups, the odds of subsequent viremia was higher in pilot participants compared to prospective controls (OR 1.52, 95% CI 1.07–2.15), though after further adjustment for confounders not measured in retrospective controls, the association was attenuated (OR 1.30, 95% CI 0.71–2.38]). Among women with sufficient follow-up time during the pilot to receive a 3-month viral load (*n* = 150), 87% were suppressed at enrollment and 93% after three months; among the 19 with elevated viral loads at enrollment, 17 improved by 3 months, including 4 of 7 with ≥ 1000 copies/mL who fully suppressed (Table 5).

**Table 4:** The risk of viremia 6-months after enrollment and association with pilot study participation

		Unadjusted		Adjusted, model 1		Adjusted, model 2	
	*n*/N* (% with viremia)	OR (95% CI)	*p*-value	aOR (95% CI)	*p*-value	aOR (95% CI)	*p*-value
Comparison to prospective controls							
Pilot	34/242 (14%)	1.21 (0.83, 1.76)	0.33	1.52 (1.07, 2.15)	0.02	1.30 (0.71, 2.38)	0.40
Controls	13/110 (12%)						
Comparison to retrospective controls							
Pilot	34/242 (14%)	1.25 (0.73, 2.14)	0.41	1.51 (0.83, 2.76)	0.18		
Controls	15/131 (11%)						

Model 1: adjustment variables included age, pregnancy status, number of clinic visits in the past year, history of viremia, dolutegravir-based regimen, and study site

Model 2: LASSO doubly selected adjustment variables from set 1 plus demographic and psychosocial characteristics

*12%, 11%, and 7% are missing future viral load in controls from records, prospective controls, and pilot participants, respectively

**Table 5:** Distribution of follow up viral load, stratified by baseline viral load, among 150 women with repeated measures

		3-month viral load *n* (row %) by enrollment viral load level			
Enrollment viral load	n [column %]	≤ 40	41–199	200–999	1000+
≤ 40	131 [87]	126 (96)	2 (2)	1 (1)	2 (2)
41–199	10 [7]	8 (80)	1 (10)	0 (0)	1 (10)
200–999	2 [1]	2 (100)	0 (0)	0 (0)	0 (0)
1000+	7 [5]	4 (57)	2 (29)	1 (14)	0 (0)
Total	150	140 (93.3)	5 (3.3)	2 (1.3)	3 (2.0)

## Discussion

In this implementation pilot study in Kisumu, Kenya, we found that engaging Mentor Mothers to promptly deliver GeneXpert viral load results after the clinic visit was feasible, acceptable, and took an average of one day between sample collection and return of viral load results to participants. This study adds to the emerging literature on implementation of near point-of-care virologic monitoring in sub-Saharan Africa by evaluating a feasible approach to timely result-tailored counseling without requiring patients to wait in the clinic for same-day results. Similar to other studies of point-of-care virologic monitoring [27–29] and novel approaches to engaging Mentor Mothers [30, 31] to support perinatal women, we did not detect a reduction in subsequent viremia associated with the intervention. Notably, our study was not designed to detect differences within our relatively short pilot period, non-randomized design, and small sample.

However, also similar to other studies, we did find that care providers valued having results quickly and felt that it improved their efficiency and ability to provide quality care [32, 33]. An enhanced role for Mentor Mothers with greater engagement at the community level was well received with provider and client perceived benefits to psychosocial health in Kenya [34]. In Uganda, Kabami found that through a standardized counseling protocol (from which our study leveraged the viral load graphic), peer mother engagement increased uptake of early infant HIV testing [35]. This body of work highlights the importance of the perceived benefits of high quality and personalized care, yet al.so the ongoing challenge of improving viral suppression in this population.

To our knowledge, factors associated with time required to reach patients with near point-of-care viral load results following a clinic visit have not previously been examined. Reassuringly, most maternal characteristics were not associated with times required for result return, suggesting that an unwillingness to be contacted did not drive delays in delivery of viral load results and counseling. We detected only a modest trend towards slower results return for those with a history of viremia, possibly driven by participant reluctance if adherence remained inconsistent; we also found a modest trend towards faster result return for those with viremia at the time of study enrollment, likely driven by Mentor Mother persistence given the concerning results. While samples collected at clinics that did not house the GeneXpert system took nearly twice as long to reach participants with results, on average participants at remote sites were still reached within two days. It is important to note that study staff played a key role in shuttling samples from remote facilities to the lab and in contacting Mentor Mothers with results. Thus, routine-care implementation of a courier system and communication between the GeneXpert site and remote sites would require further study. Mathematical modeling is a promising approach to informing geographic placement of GeneXpert systems to optimize efficiency and minimize costs [11, 36, 37]. 

In our training with Mentor Mothers, we found that they were already counseling women regarding the risks of low-level viremia, including subsequent elevation and transmission to the infant. They were enthusiastic about the graphical representation of antiretrovirals and virus in the blood as a counseling aid, however, nearly all results were delivered by phone and we did not measure use of the tool. This study adds to the literature discussing the important role of Mentor Mothers in HIV prevention efforts in Kenya [38, 39], and consideration of enhancement of their rolls within communities [34, 40]. While rapid result delivery may soon be facilitated by the use of mobile phone apps in Kenya [41], smartphone accessibility remains limited, and peer counseling will still play an important role in viral load result communication. Mentor Mothers are well poised to expedite this process.

This study has some limitations. First, we did not ask Mentor Mothers to report the time spent counseling or on phone calls with participants. While we know the overall time required to deliver results, we do not have an estimate of the additional effort required for the Mentor Mother role, nor the actual phone airtime required, which is important when considering reimbursement or incentives. We provided a flat reimbursement regardless of the time required. While a flat reimbursement can serve as an incentive to reach participants quickly, it does not incentivize longer discussions by phone. Still, most women were suppressed and only required quick words of reassurance. Those who were unsuppressed were typically referred to enhanced in-person services. Kenya’s Ministry of Health has experience with monetary incentives for community health workers [42] and therefore a suitable airtime reimbursement strategy is feasible. Second, we did not have a control group with a comparable baseline risk profile to those in the pilot study, nor an adequate follow-up period to assess the impact on subsequent viral load with rigor. Third, we did not interview study participants to assess their experiences with intervention and control conditions. Finally, while GeneXpert results for prospective controls were returned to patient files and no financial incentives were provided to Mentor Mothers, their engagement in enrollment of controls may have resulted in more enhanced viral load counseling compared to routine care.

## Conclusions

In conclusion, Mentor Mothers delivered near point-of-care viral load results to pregnant and postpartum women within one day on average, and reported that this approach improved their ability to provide quality care. GeneXpert systems are already widely distributed in Kenya for diagnosis of tuberculosis [43], and could be leveraged to enhance viral load testing. This low-cost approach, along with other patient-centered approaches [44], could help to improve viral load monitoring and counseling among pregnant and breastfeeding women living with HIV.

## Supplementary Information

Below is the link to the electronic supplementary material.

Supplementary Material 1

## References

[1] UNAIDS, AIDSInfo. Global data on HIV epidemiology and response. 2024. Available from: https://aidsinfo.unaids.org/

[2] Global guidance on criteria. and processes for validation: elimination of mother-to-child transmission of HIV, syphilis and hepatitis B virus. Geneva: World Health Organization, 2021 Licence: CC BY-NC-SA 3.0 IGO.

[3] Kabami J, Akatukwasa C, Kabageni S, Nangendo J, Byamukama A, Atwiine F, Mfitumukiza V, Munezero JBT, Arinaitwe E, Mutabazi A, Ssebutinde P, Musoke P, Kamya MR, Katahoire AR. I desire to have an HIV-free baby": pregnant and breastfeeding mothers’ perceptions of viral load testing and suppression in HIV care in southwestern Uganda. Discov Soc Sci Health. 2024. 10.1007/s44155-024-00120-1. 39524078 10.1007/s44155-024-00120-1 PMC11541329

[4] Gill MM, Natumanya EK, Hoffman HJ, Okomo G, Taasi G, Guay L, Masaba R. Active pediatric HIV case finding in Kenya and uganda: A look at missed opportunities along the prevention of mother-to-child transmission of HIV (PMTCT) cascade. PLoS ONE. 2020;15(6):e0233590. 10.1371/journal.pone.0233590. 32484815 10.1371/journal.pone.0233590 PMC7266341

[5] Tuthill EL, Odhiambo BC, Maltby AE. Understanding mother-to-child transmission of HIV among mothers engaged in HIV care in Kenya: a case report. Int Breastfeed J. 2024;19(1):14. 10.1186/s13006-024-00622-3. (**Epub 20240224**). 38395878 10.1186/s13006-024-00622-3 PMC10893718

[6] Bonner K, Mezochow A, Roberts T, Ford N, Cohn J. Viral load monitoring as a tool to reinforce adherence: a systematic review. J Acquir Immune Defic Syndr. 2013;64(1):74–8. 10.1097/QAI.0b013e31829f05ac. 23774877 10.1097/QAI.0b013e31829f05ac

[7] Ministry of Health, National AIDS & STI Control Program. Kenya HIV prevention and treatment guidelines. National AIDS & STI Control Program; 2022.

[8] Fonjungo PN, Lecher S, Zeh C, Rottinghaus E, Chun H, Adje-Toure C, Lloyd S, Mwangi JW, Mwasekaga M, Eshete YM, Pati R, Motsoane T, Mitruka K, Beukes A, Mwangi C, Bowen N, Hamunime N, Beard RS, Kabuje A, Nabadda S, Auld AF, Balachandra S, Zungu I, Kandulu J, Alemnji G, Ehui E, Alexander H, Ellenberger D. Progress in scale up of HIV viral load testing in select sub-Saharan African countries 2016–2018. PLoS One. 2023;18(3): e0282652. 10.1371/journal.pone.0282652e0282652. 36920918 10.1371/journal.pone.0282652 PMC10016655

[9] Drain PK, Dorward J, Bender A, Lillis L, Marinucci F, Sacks J, Garrett N. Point-of-care HIV viral load testing: an essential tool for a sustainable global HIV/AIDS response. Clin Microbiol Rev. 2019. 10.1128/CMR.00097-18. 31092508 10.1128/CMR.00097-18 PMC6589862

[10] Simeon K, Sharma M, Dorward J, Naidoo J, Dlamini N, Moodley P, Drain PK. Comparative cost analysis of point-of-care versus laboratory-based testing to initiate and monitor HIV treatment in South Africa. PLoS ONE. 2019;14(10):e0223669. 10.1371/journal.pone.0223669. 31618220 10.1371/journal.pone.0223669 PMC6795460

[11] Girdwood SJ, Nichols BE, Moyo C, Crompton T, Chimhamhiwa D, Rosen S. Optimizing viral load testing access for the last mile: Geospatial cost model for point of care instrument placement. PLoS ONE. 2019;14(8):e0221586. 10.1371/journal.pone.0221586. 31449559 10.1371/journal.pone.0221586 PMC6709899

[12] National AIDS, and STI Control Program (NASCOP)., Ministry of Health, Kenya. National Guidelines for PMTCT Peer EducationPsychosocial Support in Kenya: The Kenya Mentor Mother Program 2012 [Accessed: November 8, 2024]. Available from: http://guidelines.health.go.ke:8000/media/National_Guidelines__for__PMTCT_Peer_Education_and_Psychosocial_Support_in_Kenya_KMMP.pdf

[13] National AIDS and STI Control Programme (NASCOP). Kenya Population-based HIV Impact Assessment (KENPHIA) 2018: Final Report. Nairobi, Kenya: NASCOP; 2022.

[14] Knettel BA, Cichowitz C, Ngocho JS, Knippler ET, Chumba LN, Mmbaga BT, Watt MH. Retention in HIV care during pregnancy and the postpartum period in the option B + Era: systematic review and Meta-Analysis of studies in Africa. J Acquir Immune Defic Syndr. 2018;77(5):427–38. 29287029 10.1097/QAI.0000000000001616 PMC5844830

[15] Kabami J, Balzer L, Kagoya F, Okiring J, Nangendo J, Arinitwe E. Kamya M. Multicomponent intervention improves viral suppression for pregnant/postpartum women. CROI; 2023; Seattle, WA2023.

[16] Cohen S, Kamarck T, Mermelstein R. A global measure of perceived stress. J Health Soc Behav. 1983;24(4):385–96. 6668417

[17] Tsai AC, Scott JA, Hung KJ, Zhu JQ, Matthews LT, Psaros C, Tomlinson M. Reliability and validity of instruments for assessing perinatal depression in African settings: systematic review and meta-analysis. PLoS One. 2013;8(12):e82521. Epub 2013/12/18. doi: 10.1371/journal.pone.0082521. PubMed PMID: 24340036; PMCID: PMC3858316 member of the PLoS Medicine Editorial Board. This does not alter the authors’ adherence to all the PLOS ONE policies on sharing data and materials. 10.1371/journal.pone.0082521 PMC3858316 24340036

[18] Coates J, Swindale A, Bilinsky P. Household Food Insecurity Access Scale (HFIAS) for Measurement of Food Access: Indicator Guide (Version 3). Washington, D.C.: Food and Nutrition Technical Assistance Project, Academy for Educational Development; 2007.

[19] Harris PA, Taylor R, Thielke R, Payne J, Gonzalez N, Conde JG. Research electronic data capture (REDCap)--a metadata-driven methodology and workflow process for providing translational research informatics support. J Biomed Inf. 2009;42(2):377–81. 10.1016/j.jbi.2008.08.010 PMC2700030 18929686

[20] Harris PA, Taylor R, Minor BL, Elliott V, Fernandez M, O’Neal L, et al. The REDCap consortium: building an international community of software platform partners. J Biomed Inform. 2019;95:103208. 10.1016/j.jbi.2019.103208. 31078660 10.1016/j.jbi.2019.103208 PMC7254481

[21] Cephied, Xpert^®^. HIV-1 Viral Load [November 8, 2024]. Available from: https://www.cepheid.com/en-LV/tests/blood-virology-womens-health-sexual-health/xpert-hiv-1-viral-load.html

[22] Garcia-Smith D, Effken JA. Development and initial evaluation of the clinical information systems success model (CISSM). Int J Med Inf. 2013;82(6):539–52. 10.1016/j.ijmedinf.2013.01. 10.1016/j.ijmedinf.2013.01.011 23497819

[23] Aarons GA. Mental health provider attitudes toward adoption of evidence-based practice: the Evidence-Based practice attitude scale (EBPAS). Ment Health Serv Res. 2004;6(2):61–74. 10.1023/b:mhsr.0000024351.12294.65. 15224451 10.1023/b:mhsr.0000024351.12294.65 PMC1564126

[24] Williams RL. A note on robust variance estimation for cluster-correlated data. Biometrics. 2000;56(2):645–6. 10.1111/j.0006-341x.2000.00645.x. 10877330 10.1111/j.0006-341x.2000.00645.x

[25] Tibshirani R. Regression shrinkage and selection via the Lasso. J Royal Stat Soc Ser B (Methodological). 1996;58(1):267–88.

[26] STATA. Lasso for inference; 2024. Available from: https://www.stata.com/features/overview/lasso-inferential-methods/

[27] Patel RC, Oyaro P, Thomas KK, Basha GW, Wagude J, Mukui I, Abuogi LL. Impact of point-of-care HIV viral load and targeted drug resistance mutation testing on viral suppression among Kenyan pregnant and postpartum women: results from a prospective cohort study (Opt4Mamas). J Int AIDS Soc. 2023;26(11):e26182. 10.1002/jia2.26182. 37938856 10.1002/jia2.26182 PMC10631517

[28] Fairlie L, Sawry S, Pals S, Sherman G, Williamson D, Le Roux J, Team OS. More frequent HIV viral load testing with Point-Of-Care tests detects elevated viral load earlier in postpartum HIV-Positive women in a randomized controlled trial in two clinics in Johannesburg, South Africa. J Acquir Immune Defic Syndr. 2023;94(5):412–20. 37949444 10.1097/QAI.0000000000003295

[29] Mutambanengwe-Jacob MT, Maponga CC, Amico KR, Ngara B, Yende-Zuma N, Chawana TD, Stranix-Chibanda L. Impact of motivational enhanced adherence counseling and point-of-care viral load monitoring on viral load outcome in women on life-long ART: a randomized pilot study. AIDS Res Treat. 2022. 10.1155/2022/4887202. 36105074 10.1155/2022/4887202 PMC9467808

[30] Abuogi LL, Onono M, Odeny TA, Owuor K, Helova A, Hampanda K, Turan JM. Effects of behavioural interventions on postpartum retention and adherence among women with HIV on lifelong ART: the results of a cluster randomized trial in Kenya (the MOTIVATE trial). J Int AIDS Soc. 2022;25(1):e25852. 35041776 10.1002/jia2.25852 PMC8765560

[31] Larson BA, Tsikhutsu I, Bii M, Halim N, Agaba P, Sugut W, Sawe F. The effects of revised peer-counselor support on the PMTCT cascade of care: results from a cluster-randomized trial in Kenya (the EMMA study). BMC Infect Dis. 2023;23(1):257. 10.1186/s12879-023-08246-4. Epub 20230425. 37098468 10.1186/s12879-023-08246-4 PMC10127503

[32] Nakyanzi A, Naddunga F, Bulterys MA, Mujugira A, Wyatt MA, Kamusiime B, et al. It soothes your heart": a multimethod study exploring acceptability of point-of-care viral load testing among Ugandan pregnant and postpartum women living with HIV. Diagnostics (Basel). 2023. 10.3390/diagnostics14010072. 38201381 10.3390/diagnostics14010072 PMC10795616

[33] Qian SRW, Hassan SA, Scallon AJ, Oyaro P, Brown E, Wagude J, Patel RC. After viral load testing, I get my results so I get to know which path my life is taking me: qualitative insights on routine centralized and point-of-care viral load testing in Western Kenya from the Opt4Kids and Opt4Mamas studies. BMC Health Serv Res. 2022;22(1):1540. 10.1186/s12913-022-08593-z. 36528677 10.1186/s12913-022-08593-z PMC9758673

[34] Helova A, Onono M, Abuogi LL, Hampanda K, Owuor K, Odwar T, et al. Experiences, perceptions and potential impact of community-based mentor mothers supporting pregnant and postpartum women with HIV in Kenya: a mixed-methods study. J Int AIDS Soc. 2021;24(11):e25843. 10.1002/jia2.25843. 34797955 10.1002/jia2.25843 PMC8604379

[35] Kabami J, Kabageni S, Koss CA, Okiring J, Nangendo J, Ruhamyankaka E, Team E-SS. A Peer-Mother counseling intervention improves early infant HIV testing in rural Uganda. Pediatr Infect Dis J. 2025;44(11):1059–65. 40720828 10.1097/INF.0000000000004884 PMC12313110

[36] Wang Y, Wagner AD, Liu S, Kingwara L, Oyaro P, Brown E, Patel R. Using queueing models as a decision support tool in allocating point-of-care HIV viral load testing machines in Kisumu County, Kenya. Health Policy Plan. 2024;39(1):44–55. 10.1093/heapol/czad111. 37949109 10.1093/heapol/czad111 PMC10775219

[37] Nichols BE, Girdwood SJ, Crompton T, Stewart-Isherwood L, Berrie L, Chimhamhiwa D, Health E. Monitoring viral load for the last mile: what will it cost? J Int AIDS Soc. 2019;22(9):e25337. 31515967 10.1002/jia2.25337 PMC6742838

[38] Ligami C. Mentor mothers stopping vertical transmission in Kenya. Lancet HIV. 2023;10(7):e430-1. 10.1016/S2352-3018(23)00148-0. 37419568 10.1016/S2352-3018(23)00148-0

[39] Humphrey J, Wanjama E, Carlucci JG, Naanyu V, Were E, Muli L, Zimet G. Preferences of pregnant and postpartum women for differentiated service delivery in Kenya. J Acquir Immune Defic Syndr. 2023;94(5):429–36. 37949446 10.1097/QAI.0000000000003303 PMC10642693

[40] Matanda DJ, Zulu T, Odwe G, Okoth O, Nakuya Z. Experiences of young mothers with the uptake of Sulfadoxine-Pyrimethamine for intermittent preventive treatment of malaria in pregnancy: a cross-sectional study in the lake endemic region, Kenya. Front Glob Womens Health. 2024;5:1294893. 10.3389/fgwh.2024.1294893. Epub 20240326. 38596532 10.3389/fgwh.2024.1294893 PMC11002153

[41] Palladium, Kenya Health Managemen Information Systems. Navigating Nishauri Mobile Application. 2024. Available from: https://kenyahmis.org/knowledgebase/nishauri-application/

[42] Saran I, Winn L, Kipkoech Kirui J, Menya D, Prudhomme O’Meara W. The relative importance of material and non-material incentives for community health workers: evidence from a discrete choice experiment in Western Kenya. Soc Sci Med. 2020;246:112726. 10.1016/j.socscimed.2019.112726. 31869666 10.1016/j.socscimed.2019.112726

[43] de Necker M, de Beer JC, Stander MP, Connell CD, Mwai D. Economic and public health impact of decentralized HIV viral load testing: A modelling study in Kenya. PLoS ONE. 2019;14(2):e0212972. 10.1371/journal.pone.0212972. 30811510 10.1371/journal.pone.0212972 PMC6392277

[44] Odhiambo F, Onyango R, Mulwa E, Aluda M, Otieno L, Bukusi EA, Murnane PM. Evaluation of person-centered interventions to eliminate perinatal HIV transmission in Kisumu County, kenya: A repeated cross-sectional study using aggregated registry data. PLoS Med. 2024;21(8):e1004441. 10.1371/journal.pmed.1004441. 39146355 10.1371/journal.pmed.1004441 PMC11361728

