# Supplementary material for: Engaging Mentor Mothers in Rapid Return of Viral Load Results to Pregnant and Postpartum Women Living with HIV: An Implementation Pilot Study
Source: AIDS Behav. 2025 Dec 23;30(5):1589–98. doi: 10.1007/s10461-025-04987-2 (PMC12945440; doi:10.1007/s10461-025-04987-2)
Supplement: Supplementary file 1 — Supplementary Material 1 [file 10461_2025_4987_MOESM1_ESM.docx]

**Supplementary materials**

1. Feasibility and Acceptability questionnaire delivered to 8 Mentor Mothers 6 months after pilot study completion. Mentor Mothers unanimously agreed to all positive statements to a great extent, and disagreed with all negative statements.

| ACCEPTABILITY | AGREE/DISAGREE |
| --- | --- |
| I liked promptly delivering viral load results to help my clients | 0, Not at all  1, Somewhat  2, To a great extent |
| As a mentor mother, my prompt delivery of viral load results is not clinically useful | 0, Not at all  1, Somewhat  2, To a great extent |
| Counseling about low-level viremia, between 50–200 copies, is not clinically useful. | 0, Not at all  1, Somewhat  2, To a great extent |
| How likely would you be to promptly return viral load results if it was required by your supervisor? | 0, Not at all  1, Somewhat  2, To a great extent |
| How likely would you be to promptly return viral load results if this process was being used by colleagues who were happy with it | 0, Not at all  1, Somewhat  2, To a great extent |
| Do you think promptly returning viral load results is useful for improving patient adherence? | 0, Not at all  1, Somewhat  2, To a great extent |
| Do you feel that you understand how to conduct counselling about low-level viremia, between 50–200 copies, to improve patients’ adherence? | 0, Not at all  1, Somewhat  2, To a great extent |
| Do you think promptly returning viral load results is a good use your time? | 0, Not at all  1, Somewhat  2, To a great extent |
| FEASIBILITY |  |
| In general, the facility has supported my role, as mentor mother, to promptly return viral load results | 0, Not at all  1, Somewhat  2, To a great extent |
| I have the knowledge necessary to counsel patients about low-level viremia, between 50–200 copies. | 0, Not at all  1, Somewhat  2, To a great extent |
| Overall, I am satisfied with promptly returning viral load results. | 0, Not at all  1, Somewhat  2, To a great extent |
| I believe that promptly return viral load results helps me to improve patients’ adherence | 0, Not at all  1, Somewhat  2, To a great extent |
